# EHMTI-0107. Treatment of migraine in Croatia, year 2013

**DOI:** 10.1186/1129-2377-15-S1-G41

**Published:** 2014-09-18

**Authors:** V Vukovic Cvetkovic, S Zupanic, M Podgornik

**Affiliations:** 1 Department of Neurology, Univesity Hospital Center "Sestre Milosrdnice", Zagreb, Croatia; 2 General Practice "Krunoslav Reljanovic", General Practice "Krunoslav Reljanovic", Zagreb, Croatia; 3 University School of medicine Zagreb, University School of Medicine Zagreb, Zagreb, Croatia

## Aims

To estimate the burden and current treatment patterns of migraine in Croatia.

## Methods

This was a public health study conducted from April to June 2013. in Croatia. A questionnaire with 10 questiones was sent to 2750 E-mail addresses, randomly chosen from various internet sites.

## Results

Of 2750 sent E mails, 246 declared having migraine; 209 (85 %) were women (56.5% aged 18-45 years; 28.7 % aged 46-65 year; 37 (15 %) were men (8.1 % aged 18-45 years and 6.5 % aged 46-65 years). Up to 2 attacks per month had 59.4 %, 2-10 attacks had 38.6% and > 10 attacks had 2% of respondents. In the last 3 months, 74.4% was never absent from work because of headache, 20.7% was absent < 2 days and 4.9% was absent >3 days. Working with 50% reduced capacity declared 21.5% on < 2 days, 24% on 3-4 days and 21.6 % on > 5 days. In acute migraine attacks 71.5% used NSAIDs and simple analgesics, 9.4% triptans, 11.4% combination of drugs (NSAIDs+triptan) and 3.3% ergotamins. Two drugs per attack takes 39.4%, one drug 26.4% and >3 drugs 30.9%. Only 9.4% respondents was completely satisfied with acute drug treatment, 75% efficacy declared 33.7%, 50% efficacy 32.1% and less than 25% efficacy declared 23.6%. Prophylactic therapy (beta blockers, antidepressants or anticonvulsives) used 7.3%; 11% used magnesium, vitamins or herbal drugs.

## Conclusion

This study provides the most current available estimates of the impact and treatment patterns of migraine in Croatia. Migraine is under-treated and represents a major public health problem.

No conflict of interest.

